# Comparative evaluation of the potential impact of rotavirus versus hpv vaccination in GAVI-eligible countries: A preliminary analysis focused on the relative disease burden

**DOI:** 10.1186/1471-2334-11-174

**Published:** 2011-06-16

**Authors:** Sun-Young Kim, Steven Sweet, Joshua Chang, Sue J Goldie

**Affiliations:** 1 Center for Health Decision Science, Harvard School of Public Health, Boston MA, USA; 2 Harvard Global Health Institute, Cambridge, MA, USA

## Abstract

**Background:**

Immunization policymakers at global and local levels need to establish priorities among new vaccines competing for limited resources. However, comparison of the potential impact of single vaccination programs is challenging, primarily due to the limited number of vaccine analyses as well as their differing analytic approaches and reporting formats. The purpose of this study is to provide early insight into how the comparative impact of different new vaccines could be assessed in resource-poor settings with respect to affordability, cost-effectiveness, and distributional equity.

**Methods:**

We compared the health, economic, and financial consequences of introducing the two vaccines in 72 GAVI-eligible countries using a number of different outcome measures to evaluate affordability, cost-effectiveness, and distributional equity. We use simple static models to standardize the analytic framework and improve comparability between the two new vaccines. These simple models were validated by leveraging previously developed, more complex models for rotavirus and human papillomavirus (HPV).

**Results:**

With 70% coverage of a single-age cohort of infants and pre-adolescent girls, the lives saved with rotavirus (~274,000) and HPV vaccines (~286,000) are similar, although the timing of averted mortality differs; rotavirus-attributable deaths occur in close proximity to infection, while HPV-related cancer deaths occur largely after age 30. Deaths averted per 1000 vaccinated are 5.2 (rotavirus) and 12.6 (HPV). Disability-adjusted life years (DALYs) averted were ~7.15 million (rotavirus) and ~1.30 million (HPV), reflecting the greater influence of discounting on the latter, given the lagtime between vaccination and averted cancer. In most countries (68 for rotavirus and 66 for HPV, at the cost of I$25 per vaccinated individual) the incremental cost per DALY averted was lower than each country's GDP per capita. Financial resources required for vaccination with rotavirus are higher than with HPV since both genders are vaccinated.

**Conclusions:**

While lifesaving benefits of rotavirus and HPV vaccines will be realized at different times, the number of lives saved over each target populations' lifetimes will be similar. Model-based analyses that use a standardized analytic approach and generate comparable outputs can enrich the priority-setting dialogue. Although new vaccines may be deemed cost-effective, other factors including affordability and distributional equity need to be considered in different settings. We caution that for priority setting in an individual country, more rigorous comparisons should be performed, using more comprehensive models and considering all relevant vaccines and delivery strategies.

## Background

Several new vaccines have recently become available, including those against rotavirus, human papillomavirus (HPV), and pneumococcus. To set new policies for optimum use of these vaccines in different settings, participating entities including international agencies (e.g., World Health Organization [WHO]), domestic policymakers, financing coordination mechanisms (e.g., GAVI Alliance), and donors [1,2] will need new information on which to base their decisions.

Unfortunately, given the complexity of many vaccine-preventable diseases [3], the impact and inevitable tradeoffs associated with a particular vaccine program are never fully known until well after implementation. To help resolve this uncertainty for policymakers, a decision analytic approach using a disease-specific model can be used to organize, synthesize and integrate the available clinical, epidemiologic, and economic information and to estimate the cost-effectiveness of a prospective vaccine program [4,5]. Furthermore, such model-based analyses can also be useful for generating other outcomes such as intermediate (e.g., infections averted and cases averted) and long-term outcomes (e.g., deaths prevented and disability-adjusted life years [DALYs] averted) as well as the timing of those outcomes (e.g., immediate, within a few years, or decades later). Finally, a model-based analysis can help to identify the more influential determinants of a vaccine's expected impact and value in a given setting.

Recently, economic evaluation studies using a modeling approach have reported that many of the new vaccines would substantially reduce disease-specific mortality and provide good value for money in low- to middle-income country settings [6-9]. However, these vaccines also tend to be far more expensive than the traditional childhood vaccines previously introduced into these countries, many of which now face growing populations and unprecedented budget constraints. It is in this context that immunization policy makers at both global and local levels need to establish priorities among the new vaccines competing for limited resources [10,11]. Even for countries wishing to adopt multiple new vaccines, there are inevitable questions about which programs should be implemented earlier and whether some should be implemented more aggressively than others. Such priority decisions would entail various considerations (e.g., cost-effectiveness, affordability, and equity), and there has been an increasing attention paid to how best to achieve a balance between these considerations [12,13].

However, comprehensive comparison of prospective vaccine programs in low- to middle-income country settings is difficult. There have been few economic evaluations of new vaccines in resource-poor settings, for which important data is often lacking. Additionally, the cost-effectiveness profiles derived for individual vaccines from separate studies make for problematic comparison, as the analyses use different data sources and assumptions. Furthermore, independent analyses conducted for a single vaccine are often presented in different formats making it more challenging to compare results.

Nevertheless, since policymakers will likely have to make early and comprehensive priority decisions in the context of incomplete information, even a preliminary comparison based on the available data would be useful to inform deliberative dialogue among stakeholders [14]. Indeed, there recently has been an increasing demand for this type of information among international agencies and immunization financing mechanisms.

Motivated to provide early insight into how the comparative impact of different new vaccines could be assessed across different resource-poor settings (where most benefits from the vaccines would be realized), we leverage our previously conducted analyses for rotavirus and HPV (conducted for each disease separately) [6,15-19]. Given that new vaccine prices are often unknown at the early stages of vaccine arrival, and that data on country-specific program delivery costs are highly uncertain and rarely available in resource-poor settings, we take a composite cost approach that assumes the same level of plausible, collective program costs across the countries for each of the two vaccines. We thus compare the health, economic, and financial impact of introducing these two vaccines in 72 GAVI support-eligible low-income countries, with a primary focus on relative disease burden. In doing so, we standardize a simple modeling approach and analytic framework in order to facilitate comparison. We also identify and highlight methodologic challenges and influential uncertainties that should be considered as discussions about vaccine prioritization unfold.

A brief summary of the key features of the diseases caused by the two pathogens--rotavirus and HPV--and the characteristics of the two new vaccines follows:

### Rotavirus

Rotavirus is the major cause of severe, dehydrating diarrhea among children under 5 years of age [20,21]. Rotavirus infection is responsible for more than 2 million hospitalizations and approximately 527,000 deaths annually on a global level. While almost all children experience rotavirus infection by the age 5 in both developing and developed countries, the burden of disease is disproportionately high in developing countries, with ~90% of rotavirus associated deaths occurring in developing nations [20]. Human rotavirus comes in several strains, with the serotypes determined by an independent combination of two different groups of proteins, G and P. The most prevalent serotype worldwide is G1P[8], and approximately 90% of human rotavirus infections are caused by one of five types of strains: G1P[8], G2P[4], G3P[8], G4P[8], and G9P[8] [22]. The distribution of serotypes is reported to vary across countries and even local regions or over time in the same country [23]. Recently two new rotavirus vaccines (Rotarix^® ^and RotaTeq^®^) have been licensed. Rotavirus vaccines have been assigned a high priority on the global vaccine agenda, given their potential to contribute to improving general development as well as childhood health in developing countries. Based on new evidence on vaccine safety and efficacy from recent clinical trials in African settings [24], the WHO has released a global recommendation that all countries include infant rotavirus vaccination in their national immunization programs, and the GAVI Alliance has promised to provide financial support for rotavirus vaccination programs to developing countries.

### HPV

Cervical cancer is the third most common cancer in women globally, and the most common cancer in women in Eastern Africa, South-Central Asia, and Melanesia [25]. Globally, more than 500,000 cases of cervical cancer and ~270,000 deaths occur each year. Cervical cancer is also a disease of inequity, with ~80% of deaths occurring in developing countries [26]. HPV is the primary cause of cervical cancer, and HPV types 16 and 18 are known to cause ~70% of cervical cancer [27]. Worldwide, the eight most common genotypes (HPV 16, 18, 45, 31, 33, 52, 58, and 35) account for 90% of cases of cervical cancer [28]. Regional variations in cervical cancer incidence are due to differences in underlying prevalence of high-risk HPV types as well as marked differences in the availability and effectiveness of cervical screening and treatment programs. Recently, two new vaccines against HPV 16 and 18 (Gardasil^® ^and Cervarix^®^) have been made available. Like rotavirus vaccines, HPV vaccines have received a high priority and there have been global efforts to accelerate the introduction of the vaccines in developing countries. HPV vaccines are included on the list of the United Nations prequalified vaccines [29], and the GAVI Alliance has declared HPV vaccines as one of the priorities for GAVI countries [30].

**Figure 1:**
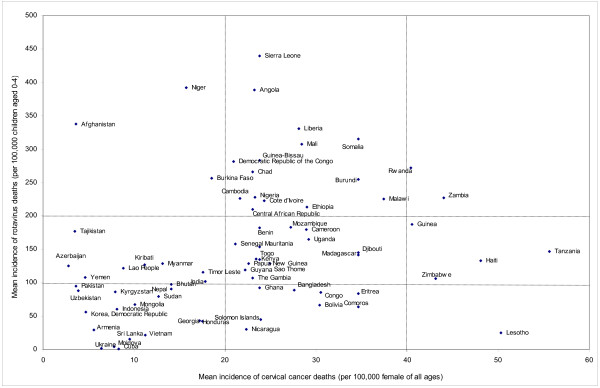
**Joint distribution of disease burden caused by rotavirus versus HPV in the 72 GAVI-eligible countries**.

Figure 1 shows the relative magnitude of the disease burden associated with rotavirus versus HPV infection.

## Methods

### Previous studies for HPV

We have previously described our series of cervical cancer models that include an individual-based stochastic model to simulate cervical carcinogenesis associated with all high-risk HPV types and a dynamic model to simulate sexual transmission of HPV 16 and 18 infections between males and females, which have been applied to multiple settings [15,31-36]. We used a likelihood-based approach to calibrate the stochastic models to empirical data, such as age-specific prevalence of HPV, age-specific incidence of cervical cancer, and HPV type distribution in normal females, cancer precursors, and cervical cancer [31]. Our empirically-calibrated models included 8 GAVI-eligible countries (2 countries in Asia, India and Vietnam [Hanoi and Ho Chi Minh City], and 6 countries in Africa [Zimbabwe, Tanzania, Nigeria, Kenya, Uganda, and Mozambique]) and 7 countries in Latin America and the Caribbean (Brazil, Argentina, Chile, Colombia, Costa Rica, Mexico, and Peru).

**Figure 2:**
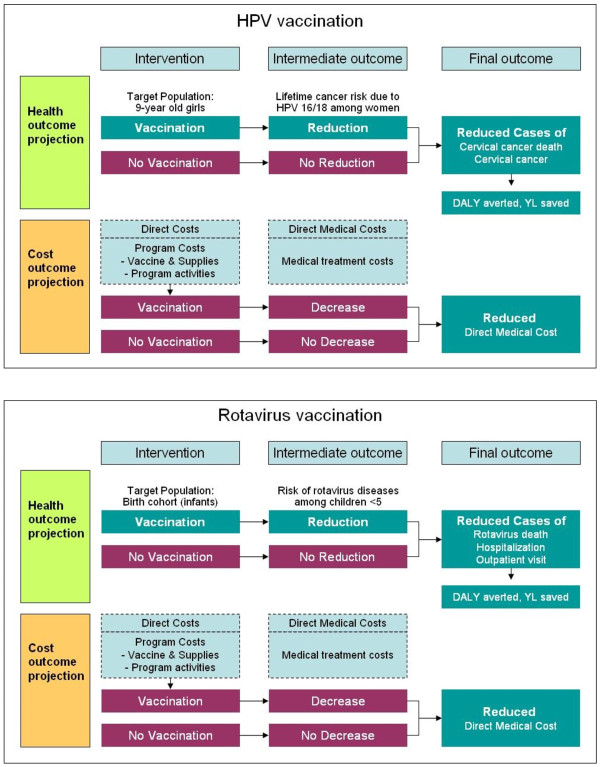
**Schematic of the companion models**.

Recognizing that the data required for such complex models are not available for all 72 GAVI-eligible countries, we constructed a companion Excel-based model (Microsoft^® ^Excel 2003 and Visual Basic for Applications 6.3). The companion model was structured as a static cohort simulation model [6,18,19,37] (Figure 2, Upper Panel), and employed simplifying assumptions relying on insights from the more complex model (e.g., the plausible ranges of parameters and the effects of simplified natural history on model outcomes) [6,18,19]. We leveraged our empirically-calibrated models [15,33,34] to validate the companion model by comparing projected model outcomes for selected countries to those obtained using the companion model.

Using the validated companion model, we synthesized population-level data on demographic structure, country-specific disease burden (incidence of cervical cancer), medical utilization patterns, and costs (expressed in 2005 international dollars [I$]) in order to estimate the health and economic consequences associated with HPV vaccination in the GAVI countries [6]. The sources of the key data are presented in Table 1[6,15,18,19,28,38-55]. Because the vaccine prices and vaccination program costs in GAVI-eligible countries are not yet known, we used a composite cost approach, assuming I$10, I$25, or I$50 for delivering a full course of HPV vaccines, based on the assumed per-dose price of the vaccines (see previous studies [6,15,18,19,32-34] for details). The model followed a single 2007 cohort of 9-year-old girls (i.e., pre-adolescent girls) over their lifetime. The model projected clinical outcomes including prevented cases of cervical cancer and cervical cancer deaths, years of life saved, and DALYs averted. The cost-effectiveness was measured in incremental cost per DALY averted from the (limited) societal perspective, and both future costs and disability-adjusted and unadjusted life years were discounted at 3% annually [44,55,56].

**Table 1:** Comparison of the two analyses using an Excel-based model: Rotavirus versus HPV [6,15,18,19,38-55]

Main features/Assumptions	Rotavirus	HPV
***Study design overview***		

Country/Region	72 GAVI-eligible countries	Same

Study type	Cost-effectiveness analysis	Same

Perspective	(Limited) societal perspective	Same

Currency	2005 international dollars (I$)	Same

Base year for discounting	2009	Same

Year of intervention	2010	Same

Primary outcome measure	ICER (I$/DALY averted)	Same

Discount rate (base-case)	Health outcome: 3%, Cost: 3%	Same

***Model type and structure***		

Model type	Static cohort model (implicitly based on a decision tree)	Same

Model outcomes	Costs Cases of rotavirus-associated deaths, hospitalizations, and outpatient visits Life years saved DALYs averted	Costs Cases of cervical cancer and cervical cancer deaths Life years saved DALYs averted

Time horizon (span)	5 years (ages 0-4)	Lifetime (ages 9-99)

Software for programming	Microsoft Excel and VBA	Same

***Assumptions on intervention and vaccine efficacy***		

Vaccine type	Rotarix^® ^or Rotateq^®^ (non-distinguished)	Gardasil^® ^or Cervarix^®^ (non-distinguished)

Strategies	Routine versus no vaccination	Same

Target population	Infants	9-year-old girls

Vaccination schedule	2,4,and 6 months of age	The second and third doses administered 1 and 6 months after the first dose

Coverage (base-case)	70%	Same

Vaccine efficacy (serotype-specific)	G1P[8]: 87% G3P[8]: 90% G4P[8]: 93% G9P[8]: 84% G2P[4], other combination:71%	100% against cervical cancer caused by HPV 16/18

Vaccine efficacy adjusted for serotype distribution	Yes	Yes

***Assumptions on natural history***		

Serotype distribution	Country-specific	Same

Duration of vaccine immunity	5 years (ages 0-4)	Lifetime (ages 9-99)

Waning of vaccine-acquired immunity	No	No

Natural immunity considered	No	No

Herd immunity considered	No	No

***Assumptions on resource use***		

Range of costs included	Direct medical costs (composite program costs and medical treatment costs)	Same

Working definition and description of composite program costs	A composite vaccination program cost was defined to be a total cost per vaccinated individual for delivering a full course of vaccines, and was assumed to include the following cost items: vaccine purchase, vaccine wastage, freight and insurance, administrative cost, immunization support (including cold chain, training, and operational costs), and other programmatic costs (including surveillance and monitoring and social mobilization). For a composite cost of I$10 and I$25, vaccine purchase cost was US$4.5 (3 doses at US$.50 each) and US$15 (3 doses at US$5 each), respectively.	Same

Levels of composite costs used in a comparative simulation	I$10 and I$25	Same

Medical utilization for treatment	1) Rotavirus gastroenteritis requiring outpatient visit: one time outpatient clinic visit 2) Rotavirus gastroenteritis requiring hospitalization: one time outpatient visit plus a 3-day admission 3) Rotavirus gastroenteritis leading to deaths: one time outpatient visit plus a 3-day admission	Stage-specific treatment costs assume diagnostic workup, inpatient and outpatient visits, follow-up [6,15]

Access to care	100% for the base-case (varied in a sensitivity analysis)	Same

***Sources of key data***		

Population prospect	UN Population Prospect, The 2006 Revision [38]	Same

Life expectancy	WHO life tables (year 2006) [39]	Same

Incidence (rotavirus-associated deaths or cervical cancer)	WHO estimates [40] Published literature [20,21]	1) GLOBOCAN 2002 [41] 2) Cancer incidence in five continents (CI5C), vol. I-VIII [42] 3) Cancer in Africa [43]

Treatment cost data	WHO-CHOICE [44]	WHO-CHOICE [44] Published literature [6,15,18,19]

Serotype distribution	Published literature [45-54]	Published literature [28]

Disability weights	Global Burden of Diseases 1990 [55]	Same

ICER = incremental cost-effectiveness ratio, DALY = disability-adjusted life year.

Sensitivity analyses were conducted to explore the influence of both uncertain parameters and assumptions on results. In addition to cost-effectiveness, in order to provide policy makers with more practical information on financial forecasting and the overall absolute reduction in disease burden over a longer term, we extended the base case analysis by adding different *roll-out scenarios *over a 10-year period (2010-2019), varying the year of vaccine introduction, maximum achievable coverage rate, and year to full coverage across countries [6].

### Previous studies for rotavirus

For rotavirus vaccines, we took a similar path. We first developed a state-transition model of rotavirus infection, which captures detailed natural history such as the age-specific incidence, probability of asymptomatic cases, rate of reinfection, correlation between strength of natural immunity and the total number of previous infections, and waning of vaccine efficacy [16]. We used the model to re-evaluate the cost-effectiveness of rotavirus vaccines in Vietnam [16]. Given the lack of data standardized for the 72 GAVI countries, we constructed a companion Excel-based model (Figure 2, Lower Panel) using analogous methods to those used for HPV (e.g., the use of a composite cost approach), and similarly validated the model by comparing the model predicted outcomes with those from more complex state-transition models [17]. We then performed similar analyses to evaluate the cost-effectiveness of rotavirus vaccines and to project reduction in burden of disease and financial requirements associated with rotavirus vaccination using a variety of scale-up scenarios. Details of model input, assumptions, and results have been described elsewhere [17].

### Comparative evaluation

While the two companion models for HPV and rotavirus [6,17] share similar model structure and many other features, not all the analytical choices are the same, as the models were developed in independent studies performed at different times. For example, the two previous studies [6,17] were based on hypothetical cohorts in different calendar years and had different scopes of the costs considered in the base-case analyses. Accordingly, to comparatively evaluate the health and economic impact of the two new vaccines (HPV and rotavirus), we further standardized on some analytic choices and scope of the costs, but kept the same model inputs and assumptions as described in the previously published articles [6,17]. Table 1 summarizes the methodological choices, model structure, and key assumptions of the two simulations as well as sources of the key data. For a simulation estimating the vaccines' cost-effectiveness, we followed a single cohort of girls aged 9 in 2010 for HPV and a birth cohort born in the same year for rotavirus, assuming two different levels of composite program costs, I$10 and I$25 per vaccinated individual. Because we have previously forecasted financial requirements for the two vaccines separately, using a range of alternative uptake and scale-up scenarios that varied by country, we conduct only a comparative analysis here that assumes both vaccines achieve the same coverage in their respective target populations. This analysis is not intended to guide budgetary planning as a flat 70% coverage rate is not realistic; rather, it is intended to simply provide insight into the comparative magnitude of resources that would be required under favorable circumstances for each vaccine.

### Model validation

As previously stated, both companion models for HPV and rotavirus have been corroborated by comparing the model-projected outcomes with those obtained using more complex (stochastic or state-transition) models for selected low- to middle-income countries [6,17]. In doing so, we adjusted the key parameter assumptions of the companion models to closely match those from the more complex models. However, given that those complex models used in the corroboration exercises are also static and therefore inherently limited in capturing indirect herd immunity effects, we also attempted to compare the outcomes obtained using our static models with those from published dynamic models for rotavirus [57-59] and HPV [32,35,36,60-63]. Additional file 1 Table S1 presents details of the process using examples of Vietnam, Kyrgyzstan, and Brazil.

## Results

### Model validation

The corroboration exercises showed that, for both rotavirus and HPV, our results using the companion models are consistent with those obtained from more comprehensive static models (Additional file 1 Table S1). The comparison with a dynamic model of rotavirus developed in Kyrgyzstan [58] showed that our static model projected slightly fewer health benefits (in terms of lives saved and DALYs averted) presumably because our static model cannot capture herd immunity effects, but that the outcomes would be of the same order of magnitude as those from the published dynamic model [42]. Similarly, for HPV, when we compared our static model with a published dynamic model [32], the incremental cost-effectiveness ratios (ICERs) from our static model were somewhat higher compared to those from the dynamic model over the ranges of program costs examined (I$25-I$400) (as expected), but the values were of the same order of magnitude as those from the dynamic model (Additional file 1 Table S1). This suggests that our companion models would yield reasonably consistent results compared with those that might be obtained should a dynamic model be used.

### Health outcomes

Vaccinating an entire single age cohort of infants and pre-adolescent girls in the 72 GAVI-eligible countries would amount to vaccinating approximately 75.8 million infants of both genders for rotavirus and 32.5 million 9-year-old girls for HPV (Table 2). Assuming 70% coverage, the numbers of vaccinated infants and pre-adolescent girls were 53.0 million and 22.7 million for rotavirus and HPV, respectively.

**Table 2:** Health outcomes of rotavirus versus HPV vaccination among the 72 GAVI-eligible countries (following a single cohort)

Health outcome measures	Rotavirus	HPV
Target population (year 2010)	75,761,613	32,453,017
Number vaccinated	53,033,129	22,717,112
Number of lives saved per 1000 vaccinated	5.2	12.6
Deaths averted (r = 0%)	273,855	285,921
*Deaths averted by region (r = 0%) (% of total)*		
*AFR D, E*	*148,541 (54%)*	*89,524 (31%)*
*AMR A, B, D*	*1,808 (1%)*	*6,651 (2%)*
*EMR D*	*30,238 (11%)*	*12,611 (4%)*
*EUR B, C*	*3,257 (1%)*	*3,554 (1%)*
*SEAR B, D*	*85,579 (31%)*	*161,724 (57%)*
*WPR B*	*4,432 (2%)*	*11,857 (4%)*
Years of life saved (r = 0%)	15,740,674	6,030,585
Years of life saved (r = 3%)	7,121,323	1,266,029
DALYs averted (r = 0%, uniform age weight)	15,767,404	6,191,573
DALYs averted (r = 3%, uniform age weight)*	7,146,859	1,304,426
*DALYs averted by region (r = 3%, uniform age-weight) (% of total)*		
*AFR D, E*	*3,748,499 (52%)*	*436,230 (33%)*
*AMR A, B, D*	*50,093 (1%)*	*34,160 (3%)*
*EMR D*	*784,703 (11%)*	*55,724 (4%)*
*EUR B, C*	*90,541 (1%)*	*21,296 (2%)*
*SEAR B, D*	*2,350,198 (33%)*	*707,996 (54%)*
*WPR B*	*122,825 (2%)*	*49,020 (4%)*
DALYs averted (r = 0%, non-uniform age weight)	18,920,531	4,847,082
DALYs averted (r = 3%, non-uniform age weight)	8,121,119	1,148,295

r = discount rate, DALY = Disability-adjusted life year.

* Base-case

Model-projected health outcomes resulting from simulating a single age cohort address the question, "what outcomes would be expected if we vaccinated 70% of a single age group of infants and 70% of a single age group of pre-adolescent girls (e.g., all 11-year-olds today) - what outcomes would we expect if we tracked the futures of these infants and girls over their respective lifetimes?" We found that, under the base-case assumptions, rotavirus vaccines would prevent ~10.4 million outpatient visits, ~1.3 million hospital admissions, and ~274,000 cases of rotavirus-associated deaths among the vaccinated cohorts in GAVI countries over a 5-year time horizon. HPV vaccines would prevent ~360,000 cases of cervical cancer and ~286,000 cases of cervical cancer-associated deaths among the cohort followed for its lifetime. Assuming 70% coverage, the number of lives saved per 1,000 vaccinated individuals would be about 5.2 for rotavirus vaccines versus 12.6 for HPV vaccines (Table 2). Additional file 1 Table S2 presents details of the country-specific health outcomes for each of the GAVI countries.

Without discounting future health outcomes (as generally is recommended in a cost-effectiveness analysis), years of life saved were ~15.74 and ~6.03 million for rotavirus and HPV vaccines, respectively. The corresponding figures for DALYs averted (without age-weighting) were ~15.77 million for rotavirus and ~6.19 million for HPV vaccines. When we assumed 3% discount rate for the future health outcomes, DALYs averted were significantly reduced to ~7.15 million for rotavirus and ~1.30 million for HPV vaccines. Note that our base-case analyses calculated DALYs without age weighting (i.e., with a uniform age weight). When we weighted DALYs by age using the standard methods by the Global Burden of Diseases (GBD) study [38], for rotavirus vaccination, the undiscounted and discounted (3%) numbers of DALYs averted increased to ~18.92 million and ~8.12 million, respectively. For HPV vaccines, the corresponding figures decreased to ~4.85 million and ~1.15 million, respectively (Table 2).

**Figure 3:**
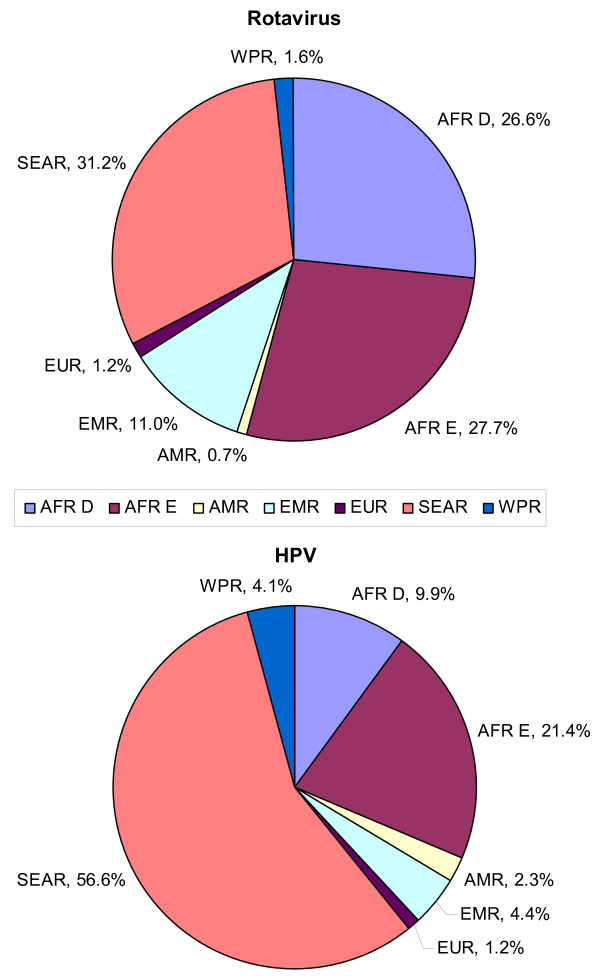
**Regional distribution of averted rotavirus-associated and cervical cancer deaths in the 72 GAVI-eligible countries**.

Figure 3 presents the distribution of the averted death burden in the GAVI-eligible countries for each of the two vaccines by region. The upper panel shows that as a result of rotavirus vaccination in the 72 GAVI countries, ~54% of the deaths averted would be in African settings and just under a third of the deaths averted would be in South Asian settings. The lower panel shows that as a result of HPV 16,18 vaccination in the 72 GAVI countries, ~57% of the deaths averted would be in South Asian settings and just under a third of deaths averted would be in African settings. Additional file 1 Figure S1 presents the age-distribution of the averted death burden projected. While the health benefits of rotavirus vaccination apply to children under age 5, the majority of the health benefits from HPV vaccination is realized in adulthood and span across decades rather than single years.

### Cost-effectiveness analysis: base-case results

**Figure 4:**
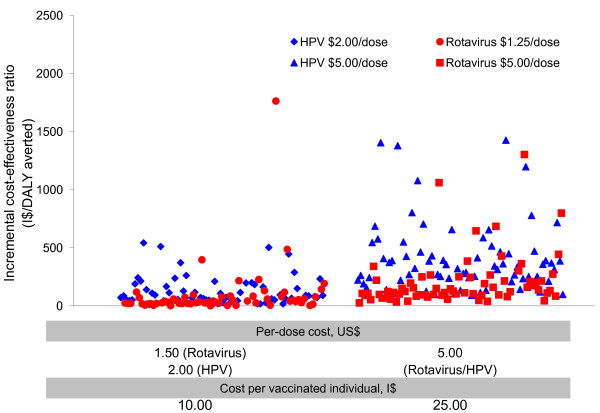
**Cost-effectiveness profiles of rotavirus versus HPV vaccination in the 72 GAVI-eligible countries**.

Figure 4 presents the cost-effectiveness analysis results of the two new vaccines for two different composite vaccination program costs, I$10 and I$25. Except for three outlier countries (Cuba, Moldova, and Ukraine) cost-effectiveness profiles of the two vaccines were very similar. In Cuba, Moldova, and Ukraine (not shown in Figure 4), ICERs of rotavirus vaccines are relatively high (I$28,480, I$12,190, and I$4,530 per DALY averted for Cuba, Ukraine, and Moldova, respectively, assuming I$25 per vaccinated child) presumably due to the relatively low rotavirus disease burden (approximately 1, 2, and 5 rotavirus deaths per 100,000 children aged less than 5 for Cuba, Ukraine, and Moldova, respectively).

For all other GAVI-eligible countries, at a cost of I$10 per vaccinated person (child or girl), for both rotavirus and HPV vaccines, in more than 60 of the 72 countries, either vaccination program was cost-saving or the cost per DALY averted was less than I$200 (which is the lowest 2007 GDP per capita expressed in 2005 I$ among the 72 GAVI countries). At a cost of I$25 per vaccinated person, the ICERs were higher, although still very attractive; using the WHO's cost-effectiveness threshold based on GDP per capita [56], rotavirus vaccines would be considered cost-saving or *very *cost-effective in 71 of 72 countries at a cost per child of I$10 and *very *cost-effective in 68 of 72 countries at a cost per child of I$25. HPV vaccines would be considered *very *cost-effective in 71 and 66 countries at a cost of I$10 and I$25 per vaccinated girl, respectively. Additional file 1 Table S3 presents details of the country-specific cost-effectiveness results for each of the GAVI countries. Additional file 1 Figure S2 shows the country-specific ICERs categorized by region, for two composite costs for each vaccine, I$10 and I$25. In general, the ICERs for rotavirus vaccine at both costs are similar for countries within the AFRO-D and AFRO-E regions. There is greater variation in country-specific ICERs for HPV vaccines within all regions, including AFRO-D and AFRO-E.

### Insight into the financial resources needed

The comparison of financial resources required for each vaccine, holding coverage and cost constant, is intended to only provide insight into the comparative magnitude of resources that would be required under favorable circumstances for each vaccine. Additional file 1 Table S4 presents the financial requirements for the first decade associated with introduction of each of the two new vaccines into GAVI countries, assuming a flat 70% coverage over a 10-year period (2010-2019). Note that the estimates of financial resources are based on the program cost per individual of I$25 but are presented in US dollars by converting the non-tradable portion of the composite costs to 2005 US dollars, since budgets are typically expressed in US dollars (or local currencies) rather than international dollars [44]. The results show that a 10-year rotavirus program would vaccinate ~537 million infants, requiring approximately US$11 billion to reach 70% of eligible infants, while a similar program for HPV would require ~US$4.9 billion to vaccinate ~237 million girls.

### Sensitivity analysis

Table 3 presents selected results of a comprehensive deterministic sensitivity analysis. Part (a) presents, using a representative example of India, selected results of univariate sensitivity analysis for a single country. For rotavirus vaccines, results (in terms of ICERs) were most sensitive to vaccination cost per child, discount rate, rotavirus disease mortality, and vaccine efficacy. For HPV vaccines, the most influential parameters included vaccination cost per vaccinated girl, discount rate, cervical cancer incidence, proportion of deaths among incident cervical cancer cases, and vaccine efficacy. For both vaccines, results were moderately sensitive to treatment costs. Part (b) of the same table shows how collective model outcomes for the GAVI-eligible countries vary as the choice of discount rate and/or type of age weight for DALY calculation are varied, for each of the two vaccines. As also described in Table 2 health outcomes in the forms of life years saved or DALYs averted were more sensitive to discount rate than to the choice of age weight. Finally, part (c) presents results of scenario analysis (or multi-way sensitivity analysis), in which vaccination costs, vaccine efficacy level, and mortality rates were simultaneously varied, in the form of numbers of countries that belong to each of the four cost-effectiveness profile categories (i.e., cost-saving, very cost-effective, cost-effective, and non-cost-effective) defined by the WHO criteria [56] based on per capita GDP. The results show that while the absolute numbers of countries that belong to each category vary widely depending on the combinations of the parameter values assumed, the relative cost-effectiveness profiles between the two vaccines (i.e., the distribution of the numbers of countries that belong to each category for each vaccine) did not vary remarkably.

**Table 3:** Selected results of sensitivity analysis

Parameters/Assumptions varied	Outcome measures	Rotavirus	HPV
***Part (a): One-way sensitivity analysis (Results for a single country, using India as a representative example)***			

Vaccination cost per individual	ICER (I$/DALY averted)		
I$5		16	saving
I$25 (base-case)		212	293
I$50		457	710
Incidence of rotavirus deaths	ICER (I$/DALY averted)		
80% of base-case		273	NA
100% (base-case)		212	NA
120% of base-case		171	NA
Incidence of cervical cancer	ICER (I$/DALY averted)		
80% of base-case		NA	429
100% (base-case)		NA	293
120% of base-case		NA	203
Ratio of hospitalization to deaths associated with rotavirus	ICER (I$/DALY averted)		
80% of base-case		215	NA
100% (base-case)		212	NA
120% of base-case		209	NA
Proportion of deaths among incident cervical cancer cases	ICER (I$/DALY averted)		
50%		NA	460
80% (base-case)		NA	293
90%		NA	261
Vaccine efficacy	ICER (I$/DALY averted)		
80% of base-case		273	397
base-case		212	293
120% of base-case		171	NA
Discount rate	ICER (I$/DALY averted)		
0%		92	saving
3% (base-case)		212	293
6%		377	1,609
Treatment costs	ICER (I$/DALY averted)		
75% of base-case		220	324
100% (base-case)		212	293
125% of base-case		204	262

***Part (b): One-way/Two-way sensitivity analysis (Aggregated results across the GAVI-eligible countries)***			

Discount rate	Years of life saved		
r = 0%		15,740,674	6,030,585
r = 3% (base-case)		7,121,323	1,266,029
r = 6%		4,025,413	323,398
Discount rate	Total incremental costs, I$		
r = 0%		1,137,130,971	66,938,177
r = 3% (base-case)		1,106,950,990	422,123,822
r = 6%		1,078,274,716	494,334,220
[Discount rate, type of age weight]	DALYs averted		
[r = 0%, K = 0]		15,767,404	6,191,573
[r = 3%, K = 0] (base-case)		7,146,859	1,304,426
[r = 0%, K = 1]		18,920,531	4,847,082
[r = 3%, K = 1]		8,121,119	1,148,295
[Discount rate, type of age weight]	No. of countries that belong to each of the cost-effectiveness categories below (at the cost of I$25 per vaccinated individual): [cost-saving, very cost-effective, cost-effective, non-cost-effective]		
[r = 0%, K = 0]		[0,71,1,0]	[23,49,0,0]
[r = 3%, K = 0] (base-case)		[0,68,4,0]	[0,66,6,0]
[r = 0%, K = 1]		[0,71,1,0]	[23,49,0,0]
[r = 3%, K = 1]		[0,68,4,0]	[0,66,5,1]

***Part (c): Scenario analysis (or multi-way sensitivity analysis) (Aggregated results across the GAVI-eligible countries)***			

Scenarios (defined by different combinations of different values of the following parameters, with other parameter values set at the base-case values): - Vaccination costs, I$10, I$25, I$50 - Vaccine efficacy, 80%-/+20% - Disease-specific mortality rates	No. of countries that belong to each of the cost-effectiveness categories below [cost-saving, very cost-effective, cost-effective, non-cost-effective]		
Scenario 1 (worst case) - Vaccination costs: I$50 - Vaccine efficacy: 80% of base-case - Mortality rates: 80% of base-case		[0,62,6,4]	[0,40,25,7]
Scenario 2 - Vaccination costs: I$25 - Vaccine efficacy: 80% of base-case - Mortality rates: 80% of base-case		[0,67,4,1]	[0,60,10,2]
Scenario 3 (base-case) - Vaccination costs: I$25 - Vaccine efficacy: base-case - Mortality rates: base-case		[0,68,4,0]	[0,66,6,0]
Scenario 4 - Vaccination costs: I$25 - Vaccine efficacy: 120% of base-case - Mortality rates: 120% of base-case		[0,69,3,0]	[1,69,2,0]
Scenario 5 (best case) - Vaccination costs: I$10 - Vaccine efficacy: 120% of base-case - Mortality rates: 120% of base-case		[5,67,0,0]	[22,50,0,0]

r = discount rate, K = 0: uniform age weight, K = 1: non-uniform age weight. DALY = disability-adjusted life year.

## Discussion

Our findings show that the number of lives saved in a head-to-head comparison of a single-age cohort of infants and pre-adolescent girls, with the rotavirus and HPV vaccines, respectively, in the GAVI countries are very similar even though the two vaccines target different populations in terms of size and gender, and even though the diseases the vaccines target have different epidemiological features. The target population for rotavirus vaccines is approximately twice as large as the target population for HPV vaccines (~76 million versus ~32 million for a single cohort) since the former includes both genders while the latter only includes girls. However, the absolute number of deaths averted within each age cohort over their lifetime is slightly lower for rotavirus vaccines than for HPV vaccines (~274,000 versus ~286,000); partly, this is due to the higher disease-specific mortality (i.e., case fatality rate) of cervical cancer compared to that of rotavirus gastroenteritis.

We found 5.2 lives were saved per 1,000 children vaccinated against rotavirus, and 12.6 lives were saved per 1000 pre-adolescent girls vaccinated against HPV 16,18. The timing of the lives saved, however, drastically differs since the deaths attributable to rotavirus occur in close proximity to the acute infection, while deaths attributable to HPV-related cervical cancer occur in the girl's adult life largely after the age of 30.

Given this time difference of lives saved, when we translated the health outcomes into DALYs averted applying a 3% discount rate, the number of DALYs averted was much higher with rotavirus vaccines compared to HPV vaccines (~7.15 versus ~1.30 million). Recently, arguments to use 'slow discounting' are made on the basis of this large time effect when the health outcome of an intervention is far in the future from the time of the actual intervention [64].

When we used WHO's cost-effectiveness threshold based on GDP per capita, in most of the GAVI countries (68 for rotavirus and 66 for HPV, at the cost of I$25 per vaccinated individual) the incremental cost per DALY averted was lower than each country's GDP per capita - implying both vaccines would meet the criterion for being "very cost-effective" [44,56,64].

We emphasize that our analysis was intended to provide a broad comparative overview of the potential impact of the rotavirus and HPV vaccines in the GAVI-eligible countries, rather than to provide precise estimates of the cost-effectiveness profiles of the two vaccines. The comparison is not based on empiric data obtained from a head-to-head clinical trial where randomization can minimize the risk of introducing bias caused by differences in the two vaccination programs. Such clinical trials are incredibly difficult to design when the comparison is based on different populations and for different conditions. Our cost-effectiveness results should be considered preliminary since the composite costs for vaccine purchase and delivery were assumed to be the same for both vaccines and uniform across all GAVI-eligible countries, and thus the differences in the cost-effectiveness results primarily reflect the relative disease burden within and across each of the GAVI countries. We therefore caution that our base-case cost-effectiveness results should be interpreted carefully. We suggest that, for priority setting decisions at a country level, more rigorous comparisons should be performed as country-specific data become available. We hope our analysis would facilitate a more specific discussion about important issues (as will be discussed later in this section) that should be considered in setting priorities among different vaccination programs competing for limited resources.

Other limitations of our analysis include the following: First, our static models are not designed to capture potential herd immunity effects that might result from either vaccine intervention, thus likely underestimating the true impact of the two vaccines. From a comparative standpoint, our results do not reflect potential differences in level of herd immunity between the two vaccines, which in turn are dependent on multiple complex factors (e.g., the nature of disease transmission and contact pattern in a given setting) for each pathogen. Second, when country-specific data were severely lacking, our analysis relied on standardized assumptions that may skew our estimates for both absolute and relative vaccine benefits in different ways for each country. For example, similar to the case of the composite vaccination program costs described previously, we relied on standardized assumptions on medical utilization practices (e.g., one-time outpatient visit plus a 3-day admission for treating severe rotavirus gastroenteritis requiring hospitalization) when calculating the costs to treat diseases caused by the two pathogens in each country. Third, due to the scarcity of relevant data on rotavirus disease in older individuals, our rotavirus model focuses on the disease burden among children under age 5 with a time horizon of 5 years. While severe rotavirus diseases occur primarily in younger children and adult cases are usually not severe, it is known that individuals older than 5 years may also develop symptomatic rotavirus diseases. Our study therefore presents a likely, but moderate, underestimate of the health benefits of rotavirus vaccination. Finally, we did not take into account the potential impact of current supplementary or alternative interventions (e.g., cervical cancer screening) on vaccine cost-effectiveness or the technological changes that may affect the cost-effectiveness profiles of the vaccines in the long-term.

Despite the limitations, the present comparative analysis, coupled with our previously published analyses, highlight some key factors related to the comparative impact, affordability, cost-effectiveness, and distributional equity that decision-makers must consider when introducing new interventions in resource-limited settings. Moreover, the analysis provides insight into several uncertainties that should be considered when assessing these vaccines.

### (a) Uncertainty about natural history, epidemiology, and vaccine efficacy

Many aspects of the natural history of rotavirus and HPV infection are still unknown, and there is still uncertainty about the vaccines' clinical effectiveness in different settings. For example, for rotavirus, additional research is needed to explore the nature of natural immunity, the serotype distribution of rotavirus in local settings, and long-term vaccine efficacy [16,65]. For HPV, in addition to uncertainty surrounding serotype distribution by setting (and over time) as well as the magnitude of cross protection, there are also uncertainties about duration of immunity and age-specific efficacy once sexual debut has occurred [4,60,66,67]. Vaccine performance, as well as natural history of HPV, in boys is less certain than in girls although information is being collected. Thus, whether an HPV vaccination program should also include boys has been a subject of much discussion [32,36,61,66,68-70]. These issues may affect the effectiveness, cost-effectiveness, and budgets of programs vaccinating against rotavirus or HPV. Accordingly, as new clinical evidence from local settings becomes available, re-evaluation of the vaccines in real-world settings will be needed.

### (b) Potential effects of different program costs

As previously discussed, it should be noted that our comparative evaluation standardizes vaccination cost per individual using a composite cost approach (i.e., set at either I$10 or I$25 for all GAVI countries) [6,17]; this is partly due to uncertainty about vaccine prices and country-specific program delivery costs associated with the introduction of either vaccine and partly for ease of comparison. Accordingly, the differences in the cost-effectiveness profiles of rotavirus versus HPV vaccines across the 72 GAVI countries stem mainly from the differences in estimated disease burden and vaccine efficacy adjusted for serotype distributions. It is of course possible that the unit prices of the two different vaccines may settle at different levels. In addition, because it is likely to be harder to reach adolescent girls than infants, the net costs for delivering the two vaccines may not be similar within some countries; on the one hand, the cost for delivering HPV vaccines could be higher, and on the other delivery may be packaged with other health programs, directed towards adolescents, a country is prioritizing [4,64,71-73]. Both of these factors could influence the prioritization of the two vaccines within a country or across all GAVI countries. Given that our sensitivity analyses show that the cost-effectiveness results of the two vaccines were most sensitive to the cost per vaccinated child or girl, it would be crucial to re-evaluate the vaccines' economic impact as more local data on delivery costs become available through country-specific data collection efforts [74,75].

### (c) Herd immunity effects

A limitation of any static companion model used to assess vaccine programs is the omission of indirect herd immunity effects that may be realized after a large-scale introduction of either rotavirus or HPV vaccination program [37,64]. Yet it is notable that the extent of herd immunity effects can vary between vaccines and across settings, depending on the scale of interventions and multiple other biological, epidemiological, and behavioral factors. A recent study in the United States has reported that a rotavirus vaccination program with 70% coverage would reduce rotavirus infection prevalence by an additional 15-25% due to herd immunity effects [57], but another recent study in Kyrgyzstan has suggested that the contribution of herd immunity effects to the overall severe rotavirus disease burden reduction would be minimal (~1%). Other studies have reported the potential herd immunity effects due to HPV vaccination would be more prominent [32,35,36,60-63]. Given that there have been no *negative *indirect effects (e.g., as shown in the case of rubella vaccine due to a shift in the age of onset of the disease) reported from either of the vaccines, the explicit inclusion of any possible herd immunity effects would presumably lead to more favorable cost-effectiveness results for both rotavirus and HPV vaccines (though the extent may vary between the two vaccines and across the countries).

### (d) Discounting

As others have discussed, a constant discount rate may underestimate the benefits of a health intervention in which benefits are realized in the far distant future when compared to an intervention where benefits occur relatively soon after implementation [4,11,64,76,77]; our comparative model-based evaluation of rotavirus versus HPV vaccines illuminates this fact clearly. Our findings show that, although the total number of averted deaths following a single cohort would be very similar between the two vaccines (~274,000 for rotavirus versus ~286,000 for HPV vaccines) under the base-case assumptions, the numbers of undiscounted DALYs averted substantially differ (~15.77 million for rotavirus versus ~6.19 million for HPV vaccines) as a majority of the deaths averted due to HPV vaccination would occur at much older ages (after age 30). This discrepancy in DALYs averted was even greater when we assumed a 3% discount rate; DALYs averted were significantly reduced to ~7.15 million for rotavirus and ~1.30 million for HPV vaccines, leading to a greater than 5-fold difference. Applying an alternative discounting technique such as a slow discounting [64,76,77] to the evaluation of HPV vaccines may lead to a more favorable cost-effectiveness profile (of an already favorable profile) of HPV vaccines. As we will discuss later, the choice of discount rate may also be related to some ethical concerns regarding whether and to what extent some lives should be considered to have more value. Accordingly, this study highlights the need for the use of alternative discount rates even in a secondary analysis, as some previous studies [11,64,76,77] recommend.

### (e) Population dynamics

In the standardized simulation for the GAVI countries, we used population data projected by the UN Population Prospects (The 2006 Revision) [38]. Among alternative projections, we chose to use the data that are based on a medium fertility scenario, incorporate background mortality, and are interpolated at 1-year intervals by age and sex. The Prospects predicts various changes in population structure over the next few decades. Most relevantly, it assumes a rapidly increasing population growth rate among adolescents and adults due to decreased overall mortality paired with relatively slow growth in new births in most developing countries (with some exceptions) [38]. For example, if we follow the trajectory of a cohort of 9-year-old girls during their lifespan, the sum of the numbers of women alive at each age between ages 40-79 years--the age band where a majority of cervical cancer deaths occur--would be ~951 million in total for the 72 GAVI countries while the corresponding figure from the snapshot in 2010 is ~372 million. In contrast, the corresponding figures for the population size of young children aged 0-4 years--the relevant age band for rotavirus vaccines--are ~371 million following the 2010 birth cohort and ~366 million using the snapshot in the same year. The remarkable increase of the age band 40-79 years explains in part the gap between the recently observed total number of cervical cancer deaths (~160,000; from a snapshot) and the model projected figure (~560,000; from a trajectory incorporating population dynamics) without HPV vaccination in the GAVI countries. This suggests that, for both vaccines, the magnitudes of the actual financial requirements and reduction in disease burden might be different from the values projected using the companion model if actual population dynamics are different from those projected by the version of population prospects that we used.

In addition to efficiency issues and uncertainties in disease burden and program costs, there are ethical as well as political considerations, that may be relevant in prioritizing new vaccine introduction in resource-poor settings. In terms of potential ethical considerations, in addition to distributional equity, our comparison of the age-distribution of the health outcomes between rotavirus and HPV vaccines suggests that, at a local level, a country's decision to place priority on one vaccine over the other under limited resources may cause public concerns of inter-generational equity. Further, at the global level, given the regional distribution of the health outcomes is different between the two vaccines (i.e., rotavirus vaccines would avert a majority of deaths in African settings while HPV vaccines would do so in South Asian settings), prioritization of one vaccine over the other may raise some inter-regional equity concerns. Finally, since rotavirus vaccination provides direct benefits to girls and boys, and right now HPV 16,18 vaccination would provide direct benefit to girls only (boys would only benefit indirectly), there could be concerns about gender equity that might be relevant to decision-making. One purposeful goal of an exercise such as this is to generate information that will stimulate a deliberative dialogue among both decision makers and stakeholders about just these very issues.

Political considerations will also be important to consider in priority setting [78]. Although a full analysis and discussion of the criteria is beyond the scope of this article, it is crucial to recognize potential policy implications of taking into account different dimensions of criteria when making decisions on which vaccines to prioritize, not only for a comparison of rotavirus versus HPV vaccines as illustrated here, but for a more comprehensive comparison including other new vaccines such as pneumococcus. Recently there has been a renewed commitment to help local policy makers with priority setting and resource allocation, arguing for the need for multi-criteria decision analysis [12,13,79]. Furthermore, when multiple new vaccines are assigned priority in a country, it would be also important to consider the implications of different timing or sequences of vaccine introduction. For example, a new vaccine that reduces early childhood mortality (e.g., rotavirus vaccine) may increase the impact of a subsequent vaccine targeting adolescents (e.g., HPV vaccine) by increasing the size of the target population for the latter vaccine. This suggests the necessity of developing a more flexible model that can capture population dynamics as well as transmission dynamics for multiple vaccines of interest.

## Conclusions

In conclusion, it would be desirable for policy makers to be able to make decisions about the timing of new vaccine introduction using country-specific information, taking into account various criteria that could affect priority-setting procedures, and considering the impact on other health sectors and systems [13,80-82]. While avertable burden and cost-effectiveness are among these criteria, other critical factors considered in decision making and prioritization include (but are not limited to) externalities, horizontal and generational equity, political criteria, and affordability. Our findings show that both rotavirus and HPV vaccines have potential to reduce mortality and be cost-effective investments in GAVI country settings, with very similar cost-effectiveness profiles in terms of cost per DALY averted. However, we note that the absolute avertable burden will vary with regional differences in disease-specific mortality, and that the comparative cost-effectiveness could very well vary with country-specific differences in programmatic delivery costs and pricing, if markedly different between the two vaccines. While the benefits of these two new vaccines will be realized at different times, the number of lives saved with these two vaccines over each target populations' lifetime will be quite similar. Our study illustrates that the use of comparable models, consistent assumptions, and standardized output that reports results similarly, allows for comparison of the two new vaccines on different dimensions; this approach can enrich the discussion about what attributes might be weighted most prominently in the priority-setting process. Nevertheless, we re-emphasize that our analysis was intended to provide an early insight on what aspects of vaccine impact should be considered in priority setting, using the examples of rotavirus versus HPV vaccines in the GAVI-eligible countries. Accordingly, for priority setting in an individual country, a more comprehensive analysis should be performed, considering all relevant vaccination programs and using country-specific data. In addition, given uncertainties surrounding the epidemiology of the diseases, the vaccines' target coverage, and the longer term vaccine effects (e.g., herd immunity effects or serotype replacement), iterative comparative evaluations will be prudent.

## Competing interests

The authors declare that they have no competing interests.

## Authors' contributions

SK and SG conceptualized and designed the study. SK and SS acquired data and performed analyses. SK, SS and SG interpreted the data and results. JC acquired data, refined the model, and performed analyses. SK and SG drafted the manuscript. SS provided administrative and technical support and provided technical expertise in managing data quality. All authors contributed to the revision of the manuscript and approved the final version.

## Pre-publication history

The pre-publication history for this paper can be accessed here:

http://www.biomedcentral.com/1471-2334/11/174/prepub

## Supplementary Material

Additional file  1 **Supplemental Appendix.** Tables S1-S4 and Figures S1-S2. Click here for file
