# Supplementary material for: Comparative evaluation of the potential impact of rotavirus versus hpv vaccination in GAVI-eligible countries: A preliminary analysis focused on the relative disease burden
Source: BMC Infect Dis. 2011 Jun 16;11:174. doi: 10.1186/1471-2334-11-174 (PMC3129299; doi:10.1186/1471-2334-11-174)

Supplemental Appendix

Accompanying the manuscript:

Comparative Evaluation of the Potential Impact of Rotavirus versus HPV Vaccination in GAVI-Eligible Countries: A Preliminary Analysis Focused on the Relative Disease Burden

Sun-Young Kim

Steve Sweet

Joshua Chang

Sue J. Goldie

Supplemental Tables and Figures

1. Table S1: Model Validation: Comparison of outcomes of companion models with those from more complex models

2. Table S2: Country-specific results: Health outcomes of rotavirus versus HPV vaccination in the 72 GAVI-eligible countries (using a single cohort)

3. Table S3: Country-specific results: Cost-effectiveness of rotavirus versus HPV vaccination in the 72 GAVI-eligible countries

4. Table S4: Impact of a 10-year (2010-2019) vaccination with rotavirus versus HPV vaccines (aggregated across the 72 GAVI-eligible countries)

5. Figure S1: Age-distribution of averted deaths in the 72 GAVI-eligible countries

6. Figure S2: Distribution of the incremental cost-effectiveness ratios for rotavirus versus HPV vaccines by region

Table S1. Model Validation: Comparison of outcomes of companion models with those from more complex models

(a) Comparison with more complex static models, using an example of Vietnam

	Rotavirus		HPV	
	Companion model (used in the present study)	Kim SY et al. [1]	Companion model (used in the present study)	Kim JJ et al. [2]
Key assumptions/analytic choices				
Currency and base year	2005 I$	2004 US$	2005 I$	2000 I$
Base year for discounting	2009	2004	2009	2007
Year of intervention	2010	2004	2010	2007
Model type	Static cohort model (implicitly based on a decision tree)	Markov model	Static cohort model (implicitly based on a decision tree)	Individual-based Monte Carlo model
Time horizon	5 years (ages 0-4)	Same	Lifetime (ages 9-99)	Lifetime (ages 9-death)
Main health outcomes	DALY averted, YLS	DALY averted, YLS	DALY averted, YLS	YLS
Vaccine type	Rotarix® or Rotateq® (non-distinguished)	Rotarix®	Gardasil® or Cervarix® (non-distinguished)	Gardasil® or Cervarix® (non-distinguished)
Strategies	Routine versus no vaccination	Same	Routine versus no vaccination	Routine vaccination, no vaccination, cervical cancer screening
Target population	Infants	Same	9-year-old girls	9-year-old girls
Vaccination schedule	2,4,6 months (3 doses)	2 and 4 months (2 doses)	The second and third doses administered 1 and 6 months after the first dose.	The second and third doses administered 1 and 6 months after the first dose.
Coverage (base-case)	70%	94%(dose 1), 93%(dose 2)	70%	70% (3 doses)
Vaccine efficacy (serotype-specific)	G1P[8]: 87%<br>G3P[8]: 90%<br>G4P[8]: 93%<br>G9P[8]: 84%<br>G2P[4], other combination:71%	G1P[8]: 91%<br>G3P[8]: 87%<br>G4P[8]: 87%<br>G9P[8]: 87%<br>G2P[4], other combination:45%	100% against cervical cancer caused by HPV 16/18	100% against infection with HPV 16 and 18
Vaccine efficacy adjusted for serotype distribution?	Yes	Yes	Yes	Yes
Serotype distribution (for Vietnam)	G1P[8]: 44.5%	Same	70.6%	71.9% (for cancer,

	Strains sharing only P[8]: 23%<br>Strains sharing only G1: 2.6%<br>Strains sharing neither antigen: 26.6%			based on data from neighboring Asian countries)
Duration of vaccine immunity	5 years (ages 0-4)	Same	Lifetime (ages 9-99)	Lifelong
Waning of vaccine-acquired immunity	No	**Yes**	No	No (base-case)<br>Sensitivity analysis: duration of protection 10-20 years
Natural immunity considered	No	**Yes**	No	**Yes**
Reinfection considered	No	**Yes (up to four)**	No	**Yes**
Herd immunity considered	No	No	No	No
Range of costs included	Direct medical costs (composite program costs and medical treatment costs)	Direct medical costs plus direct non-medical costs	Direct medical costs (composite program costs and medical treatment costs)	Direct medical costs plus direct non-medical costs
Incidence (rotavirus-associated deaths or cervical cancer)	5-year cumulative number of rotavirus deaths: 1,673 (for a 2004 cohort)	Same	Age group-specific (separate data for Hanoi and Ho Chi Minh City)	Age group-specific (separate data for Hanoi and Ho Chi Minh City)
Vaccination program costs	I$25 per vaccinated individual (vaccine price: $5 per dose)	Vaccine price: $5 per dose<br>Vaccine delivery cost: $0.7/dose	I$25 per vaccinated individual	I$10 per vaccinated individual (varied up to I$450 per vaccinated individual)
Medical utilization for treatment	1) Rotavirus gastroenteritis requiring outpatient visit: one time outpatient clinic visit<br>2) Rotavirus gastroenteritis requiring hospitalization: one time outpatient visit plus a 3-day admission<br>3) Rotavirus gastroenteritis	Same	Stage-specific treatment costs assume diagnostic workup, inpatient and outpatient visits, follow-up	Stage-specific treatment costs assume diagnostic workup, inpatient and outpatient visits, follow-up

	leading to deaths: one time outpatient visit plus a 3-day admission			
Access to care	100% for the base-case<br>(varied in a sensitivity analysis)	Same	100% for the base-case<br>(varied in a sensitivity analysis)	100%
Cost-effectiveness results				
ICERs from original base-case analyses	I$ 870/DALY averted (in 2005 I$)	$540/DALY averted (in 2004 US$)	I$420/DALY averted* (in 2005 I$)<br><br>Hanoi: I$1156/DALY averted (in 2005 I$)<br>Ho Chi Minh City: I$240/DALY averted (in 2005 I$)	Hanoi: dominated [if cytology is available]; I$650/YLS (in 2000 I$) [if only HPV screening is available]<br>Ho Chi Minh City: I$30/YLS (in 2000 I$) [if cytology is available]; I$20/YLS (in 2000 I$) [if only HPV screening is available]
ICERs after modifying key model inputs of the companion models to closely match the model inputs used in the complex models	**$530/DALY averted** (in 2004 US$)	**$540/DALY averted** (in 2004 US$)	Hanoi: **I$1,190/YLS** (in 2000 I$)**<br>Ho Chi Minh City: **I$250/YLS** (in 2000 I$)**	Hanoi: **I$1,810/YLS** (in 2000 I$)**<br>Ho Chi Minh City: **I$230/YLS** (in 2000 I$)**

HPV=human papillomavirus; DALY=disability-adjusted life year; YLS=year of life saved; ICER=incremental cost-effectiveness ratio.
* In the present analysis, for the base-case for Vietnam, we report a weighted average, assuming that nearly half of Vietnamese women face the same level of disease burden as in Hanoi and the other half as in Ho Chi Minh City.
** To improve comparability, we chose to make a comparison based on estimates from sensitivity analyses of Kim JJ et al. paper [2], which includes calculations of ICERs of vaccination compared to no vaccination (at I$25 per vaccinated girl): I$1810/YLS for Hanoi and I$230/YLS for Ho Chi Minh City.

(b) Comparison with dynamic models, using examples of Kyrgyzstan (Rotavirus) and Brazil (HPV)

	Rotavirus		HPV	
	Companion model (used in the present study)	De Balsio et al. [3]	Companion model [4]	Kim JJ et al. [5]
Key assumptions/analytic choices				
Study setting	GAVI countries (Kyrgyzstan)	Kyrgyzstan	LAC countries (Brazil)	Brazil
Currency and base year	2005 I$	NA	2005 I$	2000 I$
Base year for discounting	2009	2009	2007	2007
Year of intervention	2010	2010	2007	2007
Model type	Static cohort model (implicitly based on a decision tree)	Dynamic transmission model	Static cohort model (implicitly based on a decision tree)	Dynamic transmission model of HPV 16 and 18 infection
Time horizon	5 years (ages 0-4)	5 years	Lifetime (ages 9-death)	Lifetime (ages 12-death)
Main health outcomes	DALY averted, YLS	Case averted	DALY averted, YLS	YLS
Vaccine type	Rotarix® or Rotateq® (non-distinguished)	Rotarix® or Rotateq® (non-distinguished)	Gardasil® or Cervarix® (non-distinguished)	Gardasil® or Cervarix® (non-distinguished)
Strategies	Routine versus no vaccination	Same	Routine versus no vaccination	Routine vaccination, no vaccination
Target population	Infants	Same	9-year-old girls	12-year-old girls only<br>Girls and boys
Vaccination schedule	2,4,6 months (3 doses)	2 or 3 doses	The second and third doses administered 1 and 6 months after the first dose.	The second and third doses administered 1 and 6 months after the first dose.
Coverage (base-case)	70%	95%	70%	75%
Vaccine efficacy (serotype-specific)	G1P[8]: 87%<br>G3P[8]: 90%<br>G4P[8]: 93%<br>G9P[8]: 84%<br>G2P[4], other combination:71%	54% against severe rotavirus infections	100% against cervical cancer caused by HPV 16/18	100% against infection with HPV 16 and 18
Vaccine efficacy adjusted for	Yes	No	Yes	Yes

serotype distribution?				
Serotype distribution	G1P[8]: 32%<br>G3P[8]: 4%<br>G4P[8]: 18%<br>G9P[8]: 20%<br>G2P[4], other combination:26%	NA	68.8%	Not reported
Duration of vaccine immunity	5 years (ages 0-4)	2 months	Lifetime (ages 9-99)	Lifelong
Waning of vaccine-acquired immunity	No	**Yes**	No	No (base-case)<br>Sensitivity analysis: duration of protection 10-20 years
Natural immunity considered	No	**Yes**	No	**Yes**
Reinfection considered	No	**Yes**	No	**Yes**
Herd immunity considered	No	**Yes**	No	**Yes**
Range of costs included	Direct medical costs (composite program costs and medical treatment costs)	NA	Direct medical costs (composite program costs and medical treatment costs)	Direct medical costs plus direct non-medical costs
Incidence (rotavirus-associated deaths or cervical cancer)	5-year cumulative number of rotavirus deaths: 465 (for 2004)	221 deaths among children aged <5 years (for 2009)	Age group-specific	Age group-specific
Vaccination program costs	I$25 per vaccinated individual (vaccine price: $5 per dose)	NA	I$10-I$50 per vaccinated individual	I$25-I$400 per vaccinated individual
Medical utilization<br>(or medical treatment costs)	1)Rotavirus gastroenteritis requiring outpatient visit: one time outpatient clinic visit<br>2)Rotavirus gastroenteritis requiring hospitalization: one time outpatient visit plus a 3-day admission<br>3)Rotavirus gastroenteritis leading	NA	Stage-specific treatment costs assume diagnostic workup, inpatient and outpatient visits, follow-up<br>- Stage I: I$3,834<br>- Stages II-IV:I$3,229	Stage-specific treatment costs assume diagnostic workup, inpatient and outpatient visits, follow-up<br>- Local invasive cancer: I$5,145<br>- Regional and Distance invasive cancer: I$4,318

	to deaths: one time outpatient visit plus a 3-day admission			
Cost-effectiveness results				
ICERs from original base-case analyses	I$ 230/DALY averted<br>(in 2005 I$)	(Health benefits only)<br>Over 3 years<br>Deaths averted: 329<br>Hospitalizations averted: 5,750<br>Outpatient visits averted: 34,000	(in 2005 I$)<br>I$10/vaccinated girl<br>: cost-saving<br>I$25/vaccinated girl<br>: I$90/DALY averted<br>I$50/vaccinated girl<br>: I$400/DALY averted	(in 2000 I$)<br>I$25/vaccinated girl<br>: cost-saving<br>I$50/vaccinated girl<br>: I$130/YLS<br>I$100/vaccinated girl<br>: I$740/YLS<br>I$400/vaccinated girl<br>: I$3940/YLS
ICERs after modifying key model inputs of the companion models to closely match the model inputs used in the complex models	(Health benefits only)<br>Over 3 years<br>Deaths averted: **283**<br>Hospitalizations averted: **5,290**<br>Outpatient visits averted: **31,000**	(Health benefits only)<br>Over 3 years<br>Deaths averted: **329**<br>Hospitalizations averted: **5,750**<br>Outpatient visits averted: **34,000**	(in 2000 I$)<br>I$25/vaccinated girl<br>: **cost-saving**<br>I$50/vaccinated girl<br>: **I$260/YLS**<br>I$100/vaccinated girl<br>: **I$820/YLS**<br>I$400/vaccinated girl<br>: **I$4,190/YLS**	(in 2000 I$)<br>I$25/vaccinated girl<br>: **cost-saving**<br>I$50/vaccinated girl<br>: **I$130/YLS**<br>I$100/vaccinated girl<br>: **I$740/YLS**<br>I$400/vaccinated girl<br>: **I$3,940/YLS**

HPV=human papillomavirus; LAC=Latin America and the Caribbean; DALY=disability-adjusted life year; QALY=quality-adjusted life year; YLS=year of life saved; ICER=incremental cost-effectiveness ratio.

References

1. Kim S, Goldie SJ, Salomon JA. Cost-effectiveness of rotavirus vaccination in Vietnam. BMC Public Health 2009;9:29.

2. Kim JJ, Kobus KE, Diaz M, O'Shea M, Van Minh H, Goldie SJ. Exploring the cost-effectiveness of HPV vaccination in Vietnam: insights for evidence-based cervical cancer prevention policy. Vaccine. 2008;26(32):4015-24

3. de Blasio BF, Kasymbekova K, Flem E. Dynamic model of rotavirus transmission and the impact of rotavirus vaccination in Kyrgyzstan. *Vaccine* 2010;28:7923-7932.

4. Goldie SJ, Diaz M, Constenla D, Alvis N, Andrus JK, Kim SY. Mathematical models of cervical cancer prevention in Latin America and the Caribbean. Vaccine. 2008;26(S11):L59-72

5. Kim JJ, Andres-Beck B, Goldie SJ. The value of including boys in an HPV vaccination programme: a cost-effectiveness analysis in a low-resource setting. Br J Cancer 2007;97(9):1322–8.

Table S2. Country-specific results: Health outcomes of rotavirus versus HPV vaccination in the 72 GAVI-eligible countries (using a single cohort)

Country	Rotavirus			HPV		
	Number of rotavirus deaths averted (r=0%)	Rotavirus deaths averted (per 1000 vaccinated children)	DALYs averted (r=3%)	Number of cervical cancer deaths averted (r=0%)	Cervical cancer deaths averted (per 1000 vaccinated children)	DALYs averted (r=3%)
AFR D						
Angola	7,384	14.2	176,385	1,388	8	6,938
Benin	1,633	6.7	42,644	1,260	14	6,023
Burkina Faso	3,719	8.6	92,836	1,061	7	6,892
Cameroon	2,636	6.6	67,042	1,773	10	8,433
Chad	3,105	9.8	76,358	1,036	9	4,194
Comoros	45	2.4	1,245	199	24	1,021
Ghana	1,699	3.7	44,965	2,107	10	9,973
Guinea	1,675	6.9	43,736	1,394	15	8,562
Guinea-Bissau	535	9.5	13,602	254	14	1,414
Liberia	1,557	12.1	38,173	402	9	2,232
Madagascar	2,541	5.2	68,520	4,671	23	23,881
Mali	4,456	11.1	110,369	1,327	10	6,587
Mauritania	386	5.7	10,359	333	12	1,529
Niger	6,746	14.4	160,612	975	6	5,574
Nigeria	29,376	7.7	733,421	8,280	6	32,451
Sao Thome	16	4.7	430	15	10	65
Senegal	1,690	5.8	45,473	613	5	2,997
Sierra Leone	2,639	15.9	62,305	472	8	2,273
The Gambia	157	4.0	4,250	130	8	879
Togo	791	5.0	21,192	660	10	3,280
AFR E						
Burundi	2,689	9.3	67,442	1,880	22	8,816
Central African Republic	790	7.7	19,559	298	7	1,321
Congo	275	3.2	7,180	384	10	1,739
Cote d'Ivoire	3,272	7.6	85,210	1,602	9	8,197
Democratic Republic of the Congo	21,539	10.3	541,455	4,853	7	18,729
Eritrea	410	3.1	11,263	1,131	23	5,844
Ethiopia	16,692	7.8	443,905	16,188	19	90,553
Kenya	5,088	5.0	132,053	4,885	13	18,931
Lesotho	35	0.9	838	243	13	1,185
Malawi	3,245	8.6	81,905	2,798	18	15,106
Mozambique	3,552	6.8	90,101	2,079	9	10,190
Rwanda	2,954	9.9	75,890	2,344	25	10,314
Tanzania	5,808	5.5	146,305	11,287	27	57,742
Uganda	6,108	6.0	153,907	6,594	19	32,912
Zambia	2,372	7.8	56,160	2,242	18	11,165
Zimbabwe	923	3.6	21,407	2,365	20	8,287
AMR A, B, D						
Cuba	2	0.0	63	570	12	2,400
Guyana	35	4.8	969	69	12	455
Honduras	235	1.7	6,710	818	13	3,907
Bolivia	456	2.6	12,692	1,275	16	6,754
Haiti	961	5.2	26,275	2,896	36	15,569
Nicaragua	118	1.2	3,384	1,023	22	5,075
EMR D						
Afghanistan	10,785	12.5	254,469	820	3	4,512
Djibouti	84	5.4	2,234	180	25	873
Pakistan	11,479	3.5	315,915	5,983	5	24,929
Somalia	2,990	12.1	78,642	1,885	21	9,802
Sudan	2,432	2.9	65,913	2,747	8	11,668

Yemen	2,469	4.0	67,530	996	4	3,940
EUR B&C						
Armenia	32	1.1	907	115	11	658
Azerbaijan	510	4.7	14,086	204	6	1,131
Georgia	52	1.7	1,471	141	9	1,018
Kyrgyzstan	284	3.3	7,922	331	10	1,332
Tajikistan	880	7.1	24,217	280	5	1,861
Uzbekistan	1,472	3.6	41,188	1,161	6	7,105
Moldova	6	0.2	159	141	10	934
Ukraine	21	0.1	590	1,181	9	7,257
SEAR B&D						
Indonesia	6,718	2.3	187,993	15,730	11	81,479
Korea, Democratic Republic	464	2.1	12,982	1,316	11	6,282
Sri Lanka	119	0.6	3,424	1,240	12	4,995
Timor Leste	159	4.3	4,470	108	9	598
Bangladesh	8,482	3.4	231,585	20,104	16	94,762
Bhutan	31	3.8	858	84	19	384
India	64,776	3.6	1,777,110	114,792	14	481,097
Myanmar	2,894	4.9	79,028	4,614	16	20,434
Nepal	1,936	3.5	52,750	3,735	15	17,966
WPR B						
Cambodia	2,295	8.6	63,250	2,763	25	11,324
Kiribati	4	4.9	118	5	6	25
Lao People Democratic Republic	520	4.6	14,067	439	9	2,227
Mongolia	83	2.6	2,316	170	11	780
Papua New Guinea	602	5.0	16,493	1,256	21	6,776
Solomon Islands	18	1.7	493	111	25	596
Viet Nam	910	0.8	26,089	7,112	13	27,291

AFR=African Region; EMR=Eastern Mediterranean Region; EUR=European Region; AMR=Region of the Americas; WPR=Western Pacific Region; SEAR=South-East Asian Region; DALY=disability-adjusted life year.

Table S3. Country-specific results: Cost-effectiveness of rotavirus versus HPV vaccination in the 72 GAVI-eligible countries

Country	Rotavirus		HPV	
	ICER (I$/DALY averted) I$10 per vaccinated child	ICER (I$/DALY averted) I$25 per vaccinated child	ICER (I$/DALY averted) I$10 per vaccinated girl	ICER (I$/DALY averted) I$25 per vaccinated girl
AFR D				
Angola	saving	40	55	430
Benin	41	124	46	265
Burkina Faso	25	93	120	445
Cameroon	36	122	4	309
Chad	22	82	178	578
Comoros	132	354	saving	99
Ghana	79	228	1	300
Guinea	28	109	saving	74
Guinea-Bissau	14	75	45	236
Liberia	20	69	82	361
Madagascar	53	157	7	129
Mali	17	70	127	424
Mauritania	40	136	saving	269
Niger	15	57	217	623
Nigeria	31	106	343	1,007
Sao Thome	57	169	85	424
Senegal	44	137	233	816
Sierra Leone	6	45	213	574
The Gambia	73	209	19	289
Togo	54	164	43	339
AFR E				
Burundi	30	93	63	202
Central African Republic	30	106	180	651
Congo	96	273	104	416
Cote d'Ivoire	26	99	47	370
Democratic Republic of the Congo	27	84	299	829
Eritrea	102	273	saving	115
Ethiopia	37	107	24	160
Kenya	47	161	71	358
Lesotho	413	1077	saving	153
Malawi	33	100	70	218
Mozambique	38	122	125	446
Rwanda	12	69	saving	103
Tanzania	54	159	44	150
Uganda	38	133	saving	116
Zambia	33	112	38	203
Zimbabwe	87	259	saving	51
AMR A, B, D				
Cuba	11365	28477	70	349
Guyana	28	137	saving	176
Honduras	146	447	90	324
Bolivia	97	296	58	233
Haiti	44	146	13	89
Nicaragua	247	676	saving	120
EMR D				
Afghanistan	15	64	590	1,523
Djibouti	42	143	saving	92
Pakistan	65	215	383	1,126
Somalia	12	58	50	185
Sudan	94	276	192	637
Yemen	58	192	497	1,344
EUR B&C				
Armenia	265	723	66	301
Azerbaijan	30	141	221	699

Georgia	163	461	82	308
Kyrgyzstan	74	230	170	543
Tajikistan	24	99	265	715
Uzbekistan	5	150	194	580
Moldova	1795	4528	119	348
Ukraine	4847	12192	70	338
SEAR B&D				
Indonesia	101	323	87	350
Korea, Democratic Republic	138	383	101	381
Sri Lanka	501	1318	88	384
Timor Leste	58	178	152	436
Bangladesh	73	232	56	247
Bhutan	58	196	47	216
India	65	212	43	293
Myanmar	43	152	37	241
Nepal	76	229	70	266
WPR B				
Cambodia	3	64	45	189
Kiribati	40	147	284	766
Lao People Democratic Republic	43	160	175	497
Mongolia	94	292	152	439
Papua New Guinea	21	128	19	147
Solomon Islands	168	469	27	136
Viet Nam	264	870	133	420

I$=International dollars; DALY=Disability-adjusted life year; ICER=Incremental cost-effectiveness ratio; AFR=African Region; EMR=Eastern Mediterranean Region; EUR=European Region; AMR=Region of the Americas; WPR=Western Pacific Region; SEAR=South-East Asian Region.
* Incremental cost-effectiveness ratios are for a strategy of vaccinating 70% of a single birth cohort born in 2010 assuming a vaccination program cost of I$10 and I$25 per vaccinated child or girl compared to no vaccination.

Table S4. Impact of a 10-year (2010-2019) vaccination with rotavirus versus HPV vaccines (aggregated across the 72 GAVI-eligible countries)

Year	Rotavirus			HPV		
	Target population (million)	Number vaccinated (million)	Financial costs (2005 US$, million)	Target population (million)	Number vaccinated (million)	Financial Costs (2005 US$, million)
2010	75.8	53.0	1,108	32.5	22.7	466
2011	76.1	53.3	1,113	32.8	23.0	471
2012	76.4	53.5	1,117	33.1	23.2	475
2013	76.6	53.6	1,121	33.4	23.4	480
2014	76.8	53.8	1,124	33.7	23.6	484
2015	77.0	53.9	1,126	34.0	23.8	488
2016	77.1	54.0	1,128	34.3	24.0	493
2017	77.1	54.0	1,128	34.6	24.2	497
2018	77.1	54.0	1,129	34.9	24.4	501
2019	77.1	54.0	1,128	35.1	24.6	505
Total	767.2	537.0	11,222	338.3	236.8	4,860

* Assumed a flat coverage of 70%.

Figure S1. Age-distribution of averted deaths in the 72 GAVI-eligible countries

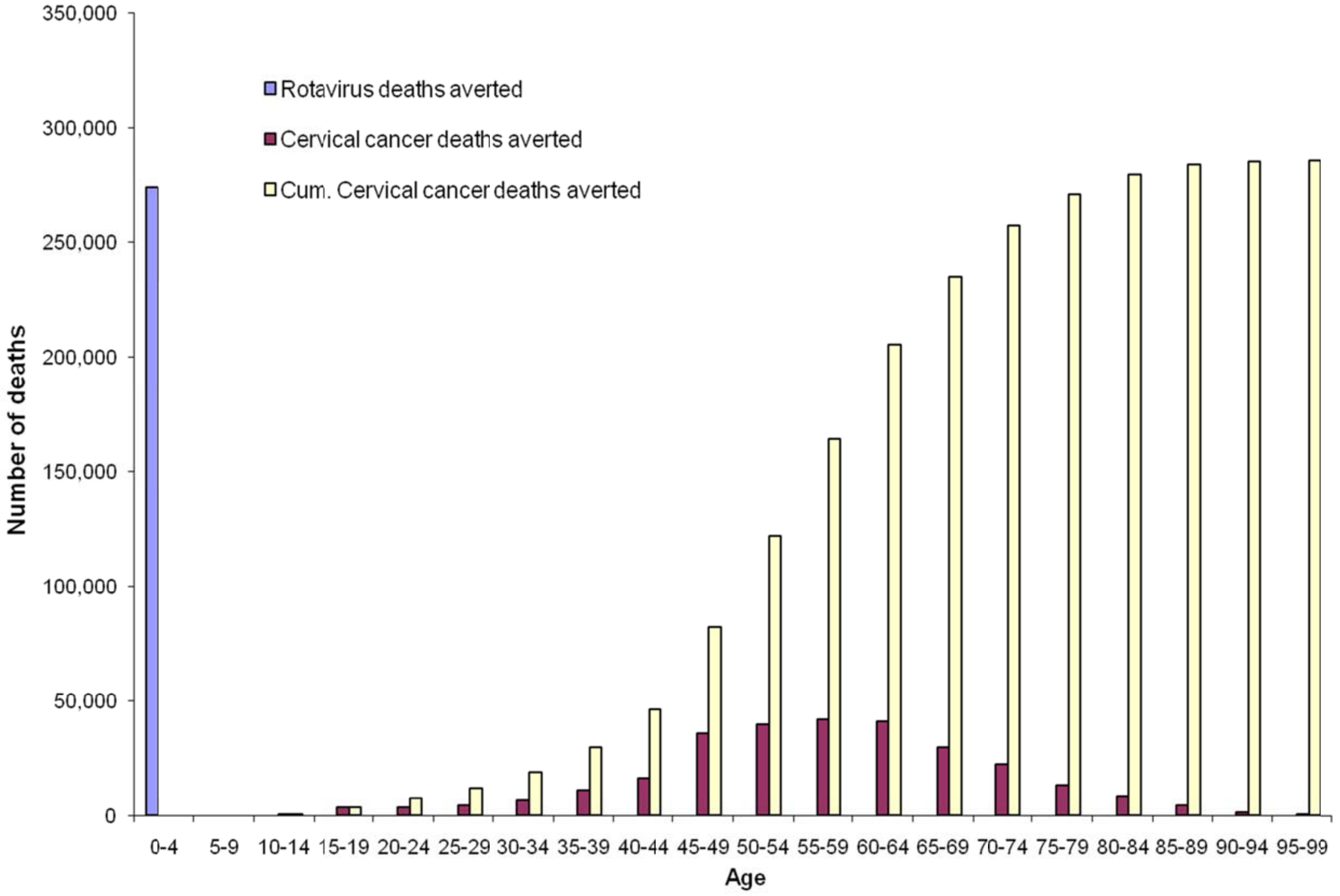

Figure S2. Distribution of the incremental cost-effectiveness ratios for rotavirus versus HPV vaccines by region

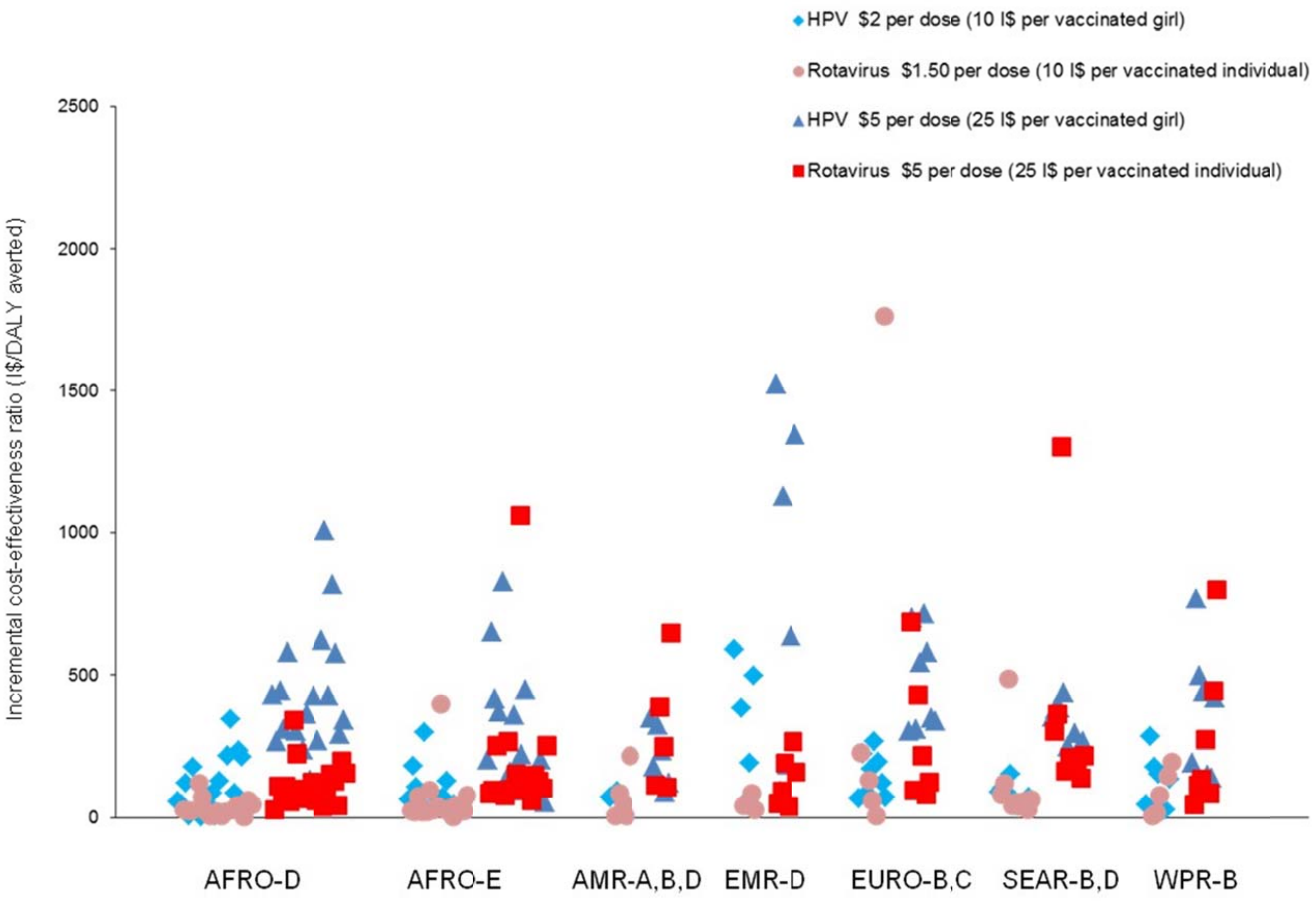

Supplement: Additional file 1 — Supplemental Appendix. Tables S1-S4 and Figures S1-S2. [file 1471-2334-11-174-S1.PDF]
